# A bibliometric study of the top 100 most-cited papers in neuroendocrine prostate cancer

**DOI:** 10.3389/fonc.2023.1146515

**Published:** 2023-03-07

**Authors:** Yu Gan, Qiangrong He, Chao Li, Bassam Lutf Mohammed Alsharafi, Hengfeng Zhou, Zhi Long

**Affiliations:** ^1^ Department of Urology, Xiangya Hospital, Central South University, Changsha, Hunan, China; ^2^ Andrology Center, Department of Urology, The Third Xiangya Hospital, Central South University, Changsha, Hunan, China

**Keywords:** bibliometric study, top 100, neuroendocrine prostate cancer, small cell prostate cancer, neuroendocrine differentiation

## Abstract

**Background:**

This study used bibliometrics to define and analyze the characteristics of the first 100 most cited papers on the topic of neuroendocrine prostate cancer (NEPC).

**Methods:**

We explored the Web of Science Core Collection database, and screened the top 100 most frequently cited articles and reviews with the title NEPC or small cell prostate cancer (SCPC). We conducted bibliometrics research on the screening results to identify the most influential journals and authors in the field of NEPC.

**Results:**

The first 100 most cited papers have been cited a total of 14,795 times, from 73 to 833 times (mean ± standard deviation, 147.95 ± 101.68). All top 100 most cited papers were published from 1984 to 2019, and the total number of citations for papers published in 2016 was significantly higher than that for papers published in other years. The journal with the largest number of published papers is “Prostate” (n=8). “Neuroendocrine differentiation” has become the most frequently used author keyword. “Oncology” is the most popular topic in the field of NEPC.

**Conclusion:**

We analyzed the first 100 most cited papers in the NEPC field by collecting detailed information, which provide guiding opinions for finding the most influential journals and authors in NEPC-related fields. We hope to help researchers and readers in this field improve their understanding of NEPC research trends and provide ideas for future research from a unique perspective.

## Introduction

Prostate cancer is one of the most common male malignancies in the world and is the most common malignancy of the genitourinary system (1). Prostatic adenocarcinoma (AD PCa) constitutes 95% of prostate cancers in pathology, and androgen-deprivation therapy (ADT) represents the first-line systemic treatment for metastatic AD PCa (2). Clinical studies have shown that ADT has a good effect on most prostate cancer patients. However, over time, prostate cancer cells eventually adapt to a low level of testosterone secondary to ADT, and develop into castration resistance prostate cancer (CRPC) (3, 4). Once prostate cancer patients enter into the CRPC stage, their median survival period is only 15–30 months. Primary anti-androgen receptor (AR) drugs, such as bicalutamide, have no significant inhibitory effect on CPRC (5). Thus, next-generation AR pathway inhibitors (ARPIs), such as enzalutamide and abiraterone, have been developed. Patients with PCa may nonetheless acquire resistance to ARPIs, whose long-term use can lead to epithelial-mesenchymal transition (EMT) and neuroendocrine differentiation (NED) in some prostate tumor cells (6–10). This type of pathology that has undergone NED is classified as neuroendocrine prostate cancer (NEPC) (11). NEPC is characterized by cancer cells proliferating and growing independently with the AR signaling pathway, and is prone to visceral metastasis and osteolytic metastasis (11–13). The overall survival period is less than one year (12).

At present, there is no effective systematic treatment plan for NEPC. Discovering ways to curb the occurrence of EMT and NED in the initial stage has attracted significant attention (14, 15). While articles on the formation of NEPC have been continuously published in recent years, it can be challenging for researchers to sort through multiple papers, as well as process and summarize huge amounts of information before classifying the papers according to their needs and interests. It is therefore necessary to conduct bibliometrics research on NEPC to facilitate researchers’ investigations in this filed.

In 1934, Paul Otlet first introduced bibliometric analysis, which aims to assess the academic influence of publications or countries on a certain topic or field, and explore the development of specific research areas (16). By using stable academic quality standards to statistically analyze published research results, bibliometrics analysis has value in guiding research trends (17, 18). Bibliometrics research has been widely used to explore research trends in various fields, such as microRNA, lncRNA, diabetes, and cancer. Considering the continuous growth of NEPC research results and the lack of published bibliometric analysis research articles in the field of NEPC, it is imperative to use quantitative methods to evaluate and analyze existing research. To a large extent, the frequency of citation can indicate the influence of an article in related disciplines and reflect the recognition of the paper by research peers. Therefore, this research used the number of citations of an article as a condition to screen the top 100 most cited papers in the field of NEPC, then conducted bibliometric research on them. The purpose of this study is to evaluate the relevant factors for the successful citation of research, which can help deepen the understanding of how NEPC-related research develops and expands. Moreover, our study can facilitate researchers’ efforts in conducting follow-up studies from different angles.

## Methods

### Research strategy

We searched the Web of Science (WoS) Core Collection database to gather studies on NEPC on December 20, 2020. The following search strategies were used: TI = (neuroendocrine prostate* OR NEPC OR small cell prostate*).

### Inclusion criteria

**Figure 1:**
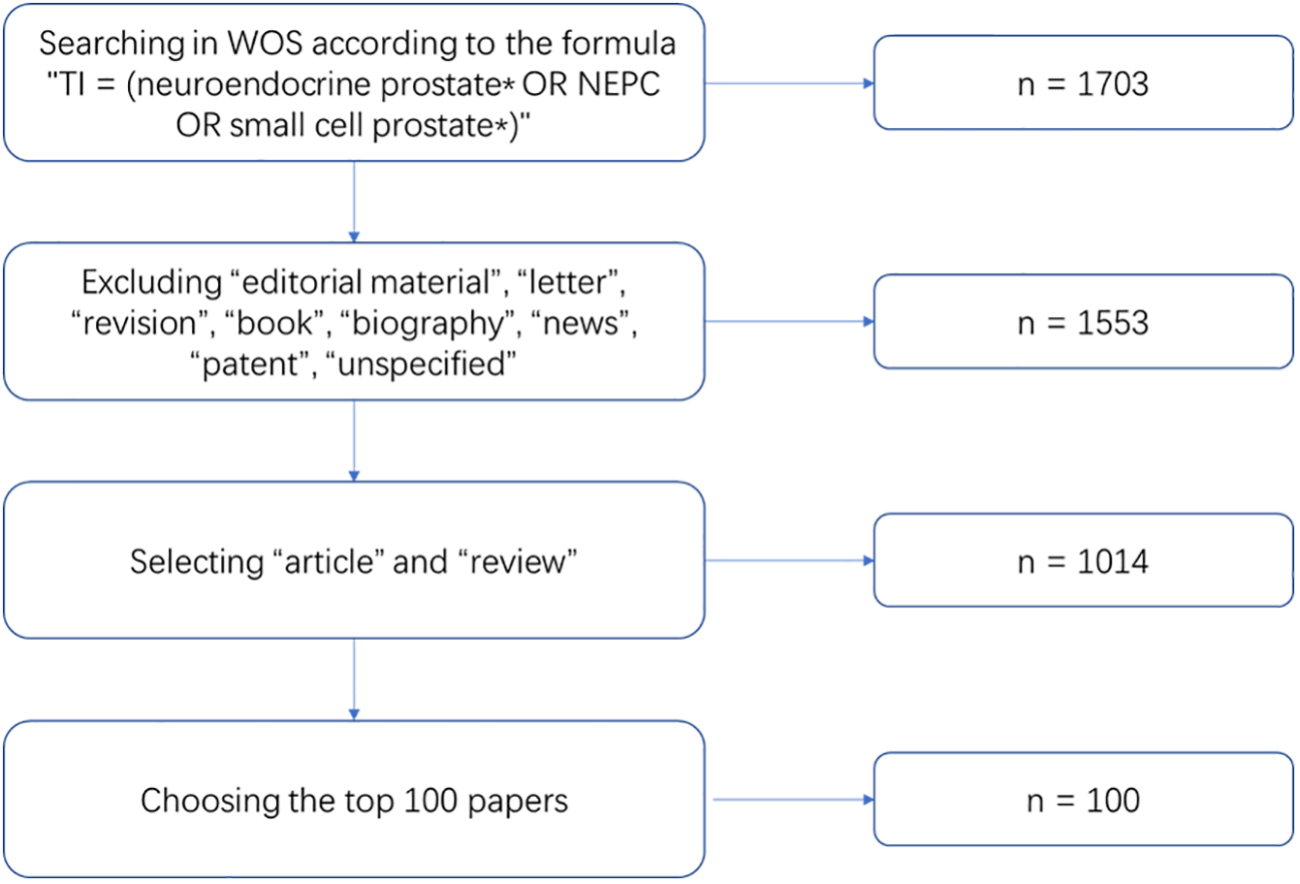
The initial search process in Web of Science (WOS).

Editorial materials, letters, revisions, books, biography, news, patent, and unspecified were excluded. Articles and reviews were targeted for screening. The selection results were listed in descending order depending on the total number of citations. We chose the top 100 most-cited papers after an independent review by two experts. The primary selection process is shown in **Figure 1**.

### Data extraction

Two authors (Yu Gan and Hengfeng Zhou) independently collected the data, and a third researcher (Zhi Long) was consulted to deal with discrepancies. The following information were collected: number of citations, journal, first author, corresponding author, country, document type, author keywords, Journal Citation Indicator (JCI) 2021, and 5-year IF. It should be noted that only the first-ranked authors were counted when there are multiple first authors. We calculated the mean and standard deviation (mean ± SD) of the number of citations.

### Statistical analysis

We used IBM SPSS Statistics for Windows, version 26.0, software to perform a one-sample t-test and simple linear regression. One-sample t-test was used to compare the difference between specific data and the mean of the population sample. Simple linear regression was used to analyze the linear correlation between the two factors. p < 0.05 was considered statistically significant.

## Results

### Citation

The top 100 most-cited papers are listed in **Table S1** in descending order based on the total number of citations. The total number of citations for the 100 papers was 14,795 times. The most cited paper in a single article was cited 833 times, and the least cited paper in a single article was cited 73 times, with an average number of citations of 147.95 ± 200.50. The most frequently cited paper was published in the journal “Nature Medicine” in 2016. The paper was written by Himisha Beltran as the first author and corresponding author. Himisha Beltran and Davide Prandi equally contributed to the work. Levi A. Garraway, Mark A., Rubin, and Francesca Demichelis jointly directed this research. This article mainly introduces the role of the evolutionary mechanism of differentiation and cloning in the evolution of NEPC.

### Publication year

**Figure 2:**
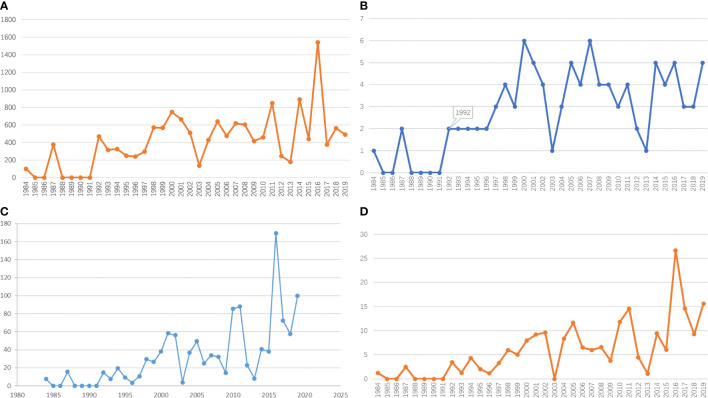
Analysis of publication year. **(A)** The relation between publication year and the number of citations. **(B)** The relation between publication year and the number of publications. **(C)** The relation between publication year and the total journal IF. **(D)** The relation between publication year and the total JCI 2021.

The top 100 most-cited papers were all published between 1984 and 2019 (**Figure 2A**). The total number of citations was the highest in 2016, with five articles published (n=1542). Among these five articles published in 2016, “Divergent Clonal Evolution of Castration-Resistant Neuroendocrine Prostate Cancer”, which was published by Himisha Beltran, made the greatest contribution (54.021%). In addition, the 5-year IF and JCI 2021 for articles published in 2016 are also the highest at 169.522 and 26.69, respectively (**Figures 2C, D**). After counting the number of articles published by year (**Figure 2B**), we can conclude that researchers have continued to publish highly cited articles in the field of NEPC since 1992. It is evident that NEPC has always attracted the attention of researchers.

### Journal

**Figure 3:**
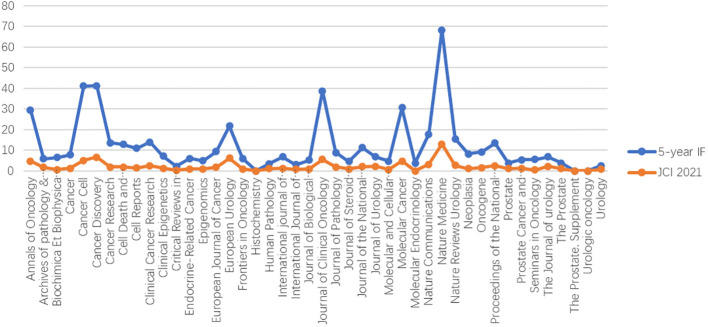
The relationship between journal IF and JCI 2021.

The top 100 most-cited papers were published in 48 journals. Eleven journals published three or more articles, and “Prostate” published the most number of articles (n =8). The 5-year IF range of these 48 journals is 2.267 to 68.311, and the JCI 2021 range is 0.31 to 13.00. JCI is a new indicator published by Clarivate to evaluate the impact of literature. JCI controls variables of different subject areas, literature types (e.g., papers, reviews), and year of publication. The resulting value represents the relative citation impact. JCI is the ratio of the influence of a paper to the global baseline; 1.0 represents the world average, and a JCI value of 2.0 means that the influence is twice the average. In order to verify the correspondence between JCI and IF, we produced a combined line chart (**Figure 3**) for the 5-year IF and JCI 2021 of the 48 journals. The trend of the two lines is roughly the same. Although JCI is a new indicator established in 2021, it can still reflect the influence of journals more objectively. When searching and evaluating journals, JCI can be used as a supplementary mark of IF.

### First author

The top 100 most-cited papers were written by 90 different authors, of which eight wrote at least two published articles. Himisha Beltran published the most number of articles (n = 4). We counted the total number of citations published and the total 5-year IF of all papers published by different authors. The top 10 authors with a total 5-year IF and the top 10 authors with the total number of citations are listed in **Table 1**.

**Table 1:** The top 10 authors with total IF and the top 10 authors with the total number of citations.

Author	Total number of citations	Number of papers	Average citation per paper	Total 5-year IF	Average 5-year IF per paper
H. Beltran	1642	4	410.5	137.49	34.37
J. I. Epstein	600	2	300.0	25.16	12.58
P. A. Abrahamsson	503	3	167.8	27.73	9.24
P. A. di Sant'Agnese	403	2	201.5	10.35	5.18
M. E. Cox	309	2	154.5	18.97	9.49
R. Aggarwal	307	2	153.5	38.79	19.49
E. Dardenne	285	1	285.0	41.16	41.16
W. Wang	269	1	269.0	7.58	7.58
H. Bonkhoff	258	2	129.0	29.61	14.81
Y. J. Bang	250	1	250.0	13.45	13.45
J. K. Lee	244	1	244.0	41.16	41.16
C. N. Papandreou	197	1	197.0	38.79	38.79
J. L. Bishop	192	1	192.0	41.23	41.23
J. Qi	182	1	182.0	41.16	41.16

### Corresponding author

Seven of the 65 corresponding authors who participated in the publication of the top 100 most-cited papers were involved in the publication of at least two articles. Huang Jiaoti participated in the publication of the most number of articles (n= 5) as a corresponding author, followed by Himisha Beltran (n = 4). The top 10 corresponding authors with total IF and the top corresponding authors with the total number of citations are listed in **Table 2**. We found that although the total number of citations of papers written by Himisha Beltran ranks first, the average number of citations is not outstanding. This is because Beltran participated in the publication of more articles as a corresponding author (n = 4) than other corresponding authors. Among these four articles, “Divergent Clonal Evolution of Castration-Resistant Neuroendocrine Prostate Cancer” has been cited as many as 833 times, far more than the other three.

**Table 2:** The top 10 corresponding authors with total IF and the top corresponding authors with the total number of citations.

·	Total number of citations	Number of papers	Average citation per paper	Total IF	Average IF per paper
Beltran, Himisha	1146	4	286.5	105.69	26.42
Epstein, Jonathan I.	600	1	600	15.16	15.16
Rubin, Mark A.	595	1	595	41.23	41.23
Jiaoti, Huang	478	5	95.6	22.29	4.46
Abrahamsson, PA	416	2	208	27.73	13.87
Aggarwal, Rahul	307	1	307	38.79	38.79
Rickman, David S.	285	1	285	41.16	41.16
Cox, ME	258	2	129	10.09	5.05
Collins, Colin C.	254	2	127	30.60	15.30
Buttyan, R	246	2	123	20.60	10.30
Witte, Owen N.	244	1	244	41.16	41.16
Papandreou, CN	197	1	197	38.79	38.79
Zoubeidi, Amina	192	1	192	41.23	41.23
Ronai, Ze'ev A.	182	1	182	41.16	41.16
Uysal-Onganer, Pinar	115	1	115	30.61	30.61
Diaz-Meco, Maria T.	86	1	86	41.16	41.16

### Country

The top 100 most-cited papers were published by authors from 10 different countries and regions. The United States has the largest number of published articles (n = 60), followed by Canada (n = 15) (**Table 3**). The number of articles published from the United States and Canada was greater than the average number of articles published (n=11) (p=0.040). This phenomenon is related to the unique distribution characteristics of NEPC. Patients with AD PCa will develop castration resistance after treatment with ADT and ARPIs, and some pathological types of patients will eventually transform into NEPC. Developed countries such as European nations and the United States are the first ones to start using AR pathway-targeted inhibitors. Their proportion of NEPC patients is also higher than that of other countries and regions. Therefore, NEPC has become a hot research topic in these regions.

**Table 3:** Countries that published the top 100 most-cited papers.

Countries	Number of publications	Total citations	Average citations per paper
USA	60	9372	156.2
Canada	15	2538	507.6
Italy	8	2441	305.125
France	6	1120	186.667
China	5	506	101.2
Japan	5	651	130.2
Germany	4	1169	292.25
Sweden	3	496	165.333
England	2	195	97.5
Spain	2	201	100.5

### Document type

The top 100 most-cited papers contain seven different article types. When the same article belongs to different article types, we repeat this article for statistics. The papers were mostly classified as an “Article” (n=64), followed by “Journal Article” (n=22), “Review” (n=21), “Research Support” (n=9), “Proceeding Paper” (n=3), “Case Report” (n=1), and “Comparative Study” (n=1). The statistics for each article type can be found in **Table 4**.

**Table 4:** Types of documents in the top 100 most-cited papers.

Type of document	Total number of citations	Number of papers	Average citation per paper
Article	9749	64	152.328
Journal article	3255	22	147.955
Review	2888	21	137.524
Research Support	846	9	94.333
Proceeding Paper	535	3	178.333
Case Report	101	1	101.000
Comparative Study	100	1	100.000

### Web of Science categories

**Figure 4:**
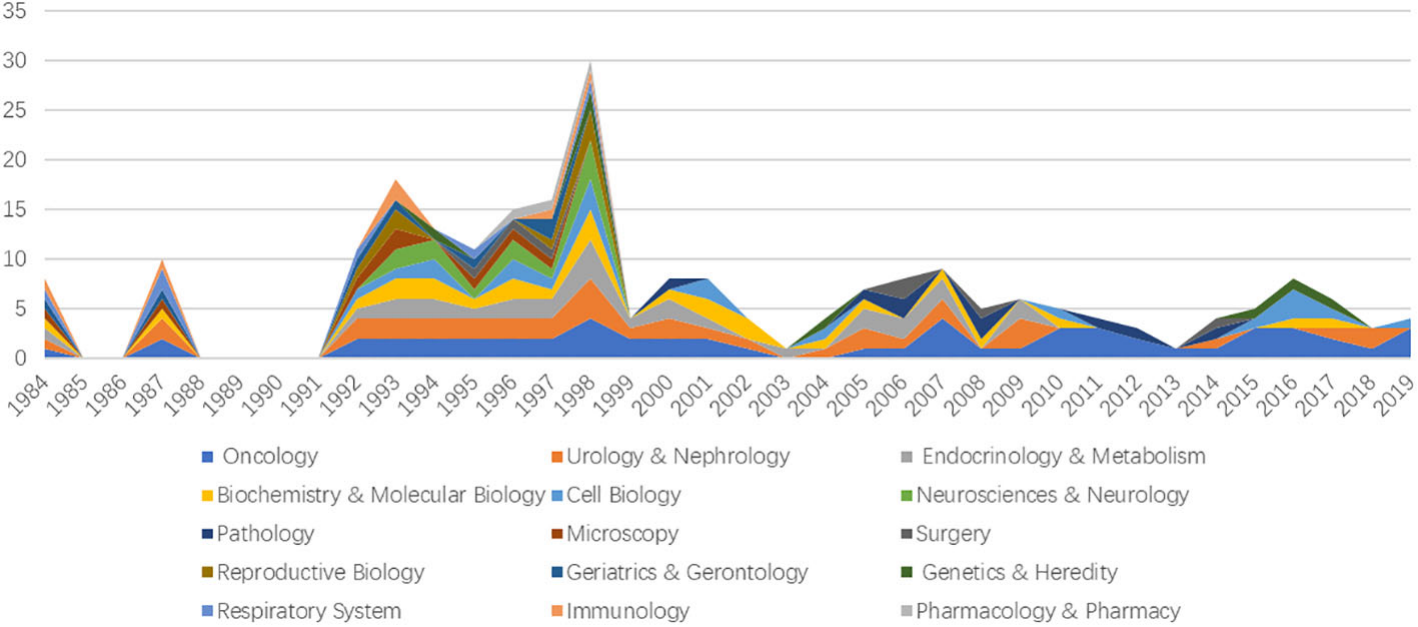
The relation between publication year and the number of publications of different WOS categories.

According to their respective research topics, the top 100 most-cited papers are divided into 15 WoS categories. Among them, “Oncology” contains the most number of articles (n =56), followed by “Urology & Nephrology” (n=37), “Endocrinology & Metabolism” (n =28), “Biochemistry & Molecular Biology” (n=26), and “Cell Biology” (n =20); the details are listed in **Table 5**. In addition, we produced **Figure 4** to show the number of papers contained in each classification field by year. As can be seen from the figure, “Neurosciences & Neurology,” “Pathology,” “Reproductive Biology,” “Reproductive Biology,” “Reproductive Biology,” “Reproductive Biology,” and “Pharmacology & Pharmacy” did not produce any new papers after 2000. Although there are many articles in “Endocrinology & Metabolism,” no frequently cited articles have been produced after 2009.

**Table 5:** WOS categories in the top 100 most-cited papers.

WOS categories	Times	Total citation times	Citations per paper	Total IF	Average IF per paper	Total JCI
Oncology	56	8455	150.982	793.627	14.172	127.18
Urology & Nephrology	37	5049	136.459	270.548	7.312	65.14
Endocrinology & Metabolism	28	3793	135.464	150.277	5.367	32.03
Biochemistry & Molecular Biology	26	4123	158.577	255.195	9.815	46.15
Cell Biology	20	3844	192.200	338.805	16.940	53.25
Neurosciences & Neurology	12	1649	137.417	73.875	6.156	16.15
Pathology	9	1513	168.111	57.202	6.356	16.26
Microscopy	8	1051	131.375	44.482	5.560	9.38
Surgery	7	1217	173.857	47.587	6.798	13.57
Reproductive Biology	7	1110	158.571	35.420	5.060	6.91
Geriatrics & Gerontology	7	988	141.143	47.827	6.832	10.42
Genetics & Heredity	7	723	103.286	56.430	8.061	11.09
Respiratory System	6	851	141.833	35.383	5.897	7.37
Immunology	6	788	131.333	40.674	6.779	7.39
Pharmacology & Pharmacy	3	393	131.000	20.332	6.777	4.80

### Author keywords

Since only 38 of the top 100 most-cited papers gave author keywords in WoS, we only included the keywords of these 38 articles in the statistics in this link. These 38 most cited articles contain 103 keywords, with “Neuroendocrine differentiation” showing the most occurrences (n = 12), followed by “prostate cancer” (n = 11). The number of occurrences of the author keywords is listed in **Table 6**.

**Table 6:** The author keywords that appear at least three papers in the top 100 most-cited papers.

Keywords	Numbers
Neuroendocrine differentiation	12
prostate cancer	11
neuroendocrine	6
small cell carcinoma	6
neuroendocrine cell	5
androgen receptor	4
immunohistochemistry	3
prognosis	3
hormone refractory	3
neuron-specific enolase	3

## Discussion

As the use of ARPIs becomes more widespread, therapy-induced NEPC—as an advanced stage of PCa—is showing a higher prevalence rate and is gradually attracting the attention of many scholars. With the gradual widespread application of pathological biopsy of metastases and immunohistochemistry in clinical diagnosis, the number of patients diagnosed with NEPC is increasing (19, 20). At the same time, as more developed countries begin using secondary generation AR pathway-targeted inhibitors such as abiraterone and enzalutamide, the diagnosis rate of NEPC in Europe, America, and other developed nations are also higher than that in developing countries. In the early stage, there have been many debates about the status and significance of NEPC and NED. With the help of technical methods such as gene sequencing and genetically engineered mice, people have gradually realized the uniqueness of NEPC in, for example, gene expression profiles and biological characteristics. Many scholars have therefore chosen to conduct research on NEPC as an independent disease.

In this paper, we selected the top 100 most-cited papers in the NEPC field from the WoS Core English Database. By classifying and counting the top 100 most-cited papers, we discuss the development history and possible future research hotspots of the NEPC.

We chose to conduct our search in the WoS Core English Database as the WoS is the world’s largest comprehensive academic information resource covering the broadest scope of disciplines. It contains the core academic journals of various university disciplines, making the data and statistical results comparable to bibliometrics research in other fields. There are some articles originating from journals that are currently out of print. The articles published in these journals can still be searched and cited normally, their inclusion in the statistics has no impact on the statistical results.

We used the number of citations as the main condition for screening articles in this research. Compared with the 5-year IF and JCI 2021, the number of citations can more truly reflect the degree to which the article is recognized by peers (21, 22). In our statistics, the number of citations for a paper ranges from 73 to 833, and the total number of citations is 14,795. The most cited article is “Divergent Clonal Evolution of Castration-Resistant Neuroendocrine Prostate Cancer.” This article proposes a differentiated cloning model of CRPC establishment. In AD PCa tissue, adenocarcinoma cells differentiate into different types of sub-clonal cells. Under the pressure of targeted inhibitors of the AR pathway, sub-clonal cells that can adapt to the environment become the main cells inside the tumor and finally complete the transformation of tumor pathological types. Experimental results and clinical phenomena show that most of the results of adaptation are transformed into NEPC. In addition, this article further proposes that the epigenetic genome and cell plasticity play an important regulatory role in the neuroendocrine differentiation process (23).

The top 100 most-cited papers were published between 1984 and 2019. Since 1994, there has been an output of highly cited articles every year. In particular, the total number of papers published throughout the year was the highest in 2016, followed by 2014 and 2011. However, the statistical results do not include articles published from 2020 to 2022, many of which have a higher 5-year IF. This may be due to the publication time being too close to the statistical time. Even for important results, it is difficult to obtain a high number of citations in a short period of time.

The 5-year IF is the other indicator we pay attention to. The IF reflects the influence of the entire journal in the field in recent years; however, the contribution of each paper in the journal is different. Among the 48 journals counted, except for three journals that do not have a 5-year IF record, the minimum IF is 2.548, which reflects how papers with more citations may be published in journals with lower IF, such as “Proposed Morphologic Classification of Prostate Cancer with Neuroendocrine Differentiation” which was published in the American Journal of Surgical Pathology in 2014. This article reclassifies the clinicopathological types of NEPC. This result will lead to more accurate targeted therapy for patients with different pathological types of prostate cancer (19). Therefore, when assessing the value of a paper, it may be more accurate to focus on the number of citations of the article. However, there is still a large correlation between the journal’s 5-year IF value and the number of citations in the paper (r=0.959, p=0.000). When searching for papers about NEPC, we recommend using 5-year IF as an auxiliary search standard.

Our statistical results suggest that the top 100 most-cited papers were written by 90 first authors and published by 65 corresponding authors. Among them, the most noteworthy is Himisha Beltran, who is the director of Clinical and Translational Research at the Englander Institute for Precision Medicine. She has published many high-quality articles on prostate cancer and was involved in the publication of papers that ranked first in both the total number of citations and the total 5-year IF statistics. Himisha Beltran participated in the publication of a total of five articles, whose number of citations is 99 to 833, and all paper types are classified as an “Article.” The most cited article is “Divergent Clonal Evolution of Castration-Resistant Neuroendocrine Prostate Cancer” published in “Nature Medicine” in 2016. Scholars who are studying prostate cancer should pay attention to papers published by Himisha Beltran.

Among the 100 most-cited papers, 64 are considered articles. It can be seen that an article is a widely recognized type of paper. Although proceeding papers showed the highest average number of citations, these three papers are still mainly articles. In addition, the majority of these 100 articles classified as “Article,” indicating that NEPC is still constantly making progress. The “Article” generally refers to the researcher’s detailed global presentation of research results. The “Review” generally refers to the researcher’s summary and review of the results of previous experiments or research results in a particular field of study. Thus, the number of papers classified as a “Review” will gradually increase when there are adequate research results.

The top 100 most-cited papers come from 15 different WoS categories. There are more than 20 articles on “Oncology,” “Urology and Nephrology,” “Endocrinology and Metabolism,” “Biochemistry and Molecular Biology,” and “Cell Biology.” The total number of citations of papers in Oncology has obvious advantages. At the same time, over time, the number of highly cited articles in Endocrinology and Metabolism, Neuroscience and Neurology, Reproductive Biology, Geriatrics and Gerontology, Respiratory System, Immunology, Pharmacology and Pharmacy has decreased significantly. This may be due to the deepening of NEPC understanding, gradually clarifying and refining the direction of NEPC research.

Excluding duplicates, 103 authors’ keywords were counted, but only 10 appear frequently. Among them, “neuroendocrine differentiation” has become the most frequently used keyword and appeared in articles from 2000 to 2015. Neuroendocrine differentiation has been confirmed to be one of the theories that promote NEPC formation, and it being a frequently cited keyword can explain why it represents an important research direction of NEPC (24, 25). In addition, although “lineage plasticity” does not appear in the statistical results, it plays a key role in the neuroendocrine differentiation model (2, 26). Therefore, we believe that lineage plasticity may attract more attention in future research.

We acknowledge that our research has several inevitable limitations. First, there have been many debates about the uniqueness and significance of NEPC and NED at the early stage. The small-cell prostate cancer (SCPC) has not been classified in the NEPC classification. Although we added “SCPC” into the select formula, it is still inevitable that some potentially critical articles may have been missed. Second, our research began on October 30, 2022, so recently published important studies that have not been cited enough may have also been missed. This may be the reason why some important articles have not been included in the statistics. The list of the top 100 most-cited papers may change over time, and we will continue to pay attention to the list and the changes in its statistical results. Third, since only 38 articles provide author keywords in the WoS, the statistical results on keywords may be biased. Fourth, our study only counted papers in the WoS. However, in different databases, the papers included and the data of each paper are not exactly consistent, which may also lead to a distortion of the results.

Despite these limitations, our research is still the first bibliometrics research on NEPC. It presents some of the authors who have made significant contributions to the field of NEPC, and summarizes the development process of NEPC research. Our study provides scholars who wish to join the field of NEPC with the direction to retrieve important literature and try to identify possible future research directions of NEPC.

## Data availability statement

The original contributions presented in the study are included in the article/**Supplementary Material**. Further inquiries can be directed to the corresponding authors.

## Author contributions

HZ, CL and QH collected the references. HZ, ZL and YG screened the papers. YG and HZ wrote the initial draft. HZ, ZL and YG reviewed and edited the initial draft. HZ and BA wrote the manuscript. YG acquired the financial support for the project leading to this publication.
