# Supplementary material for: A bibliometric study of the top 100 most-cited papers in neuroendocrine prostate cancer
Source: Front Oncol. 2023 Mar 7;13:1146515. doi: 10.3389/fonc.2023.1146515 (PMC10027713; doi:10.3389/fonc.2023.1146515)
Supplement: Supplementary file 1 [file Table_1.pdf]

Table S1. The top 100 most-cited papers

author	DOI	title
H. Beltran, D. Prandi, J. M. Mosquera, M. Benelli, L. Puca, J. Cyrta, C. Marotz, E. Giannopoulou, B. V. S. K. Chakravarthi, S. Varambally, S. A. Tomlins, D. M. Nanus, S. T. Tagawa, E. M. Van Allen, O. Elemento, A. Sboner, L. A. Garraway, M. A. Rubin and F. Demichelis	10.1038/nm.4045	Divergent clonal evolution of castration-resistant neuroendocrine prostate cancer
H. Beltran, D. S. Rickman, K. Park, S. S. Chae, A. Sboner, T. Y. MacDonald, Y. Wang, K. L. Sheikh, S. Terry, S. T. Tagawa, R. Dhir, J. B. Nelson, A. de la Taille, Y. Allory, M. B. Gerstein, S. Perner, K. J. Pienta, A. M. Chinnaiyan, Y. Wang, C. C. Collins, M. E. Gleave, F. Demichelis, D. M. Nanus and M. A. Rubin	10.1158/2159-8290.Cd-11-0130	Molecular Characterization of Neuroendocrine Prostate Cancer and Identification of New Drug Targets
J. I. Epstein, M. B. Amin, H. Beltran, T. L. Lotan, J.-M. Mosquera, V. E. Reuter, B. D. Robinson, P. Troncoso and M. A. Rubin	10.1097/pas.0000000000000208	Proposed Morphologic Classification of Prostate Cancer With Neuroendocrine Differentiation
P. A. di Sant'Agnese	10.1002/1097-0142(19920701)70:1+<254::Aid-cncr2820701312>3.0.Co;2-e	Neuroendocrine differentiation in carcinoma of the prostate. Diagnostic, prognostic, and therapeutic implications
R. Aggarwal, J. Huang, J. J. Alumkal, L. Zhang, F. Y. Feng, G. V. Thomas, A. S. Weinstein, V. Friedl, C. Zhang, O. N. Witte,	10.1200/jco.2017.77.6880	Clinical and Genomic Characterization of Treatment-Emergent Small-Cell Neuroendocrine Prostate Cancer: A Multi-institutional Prospective Study

P. Lloyd, M. Gleave, C. P. Evans, J. Youngren, T. M. Beer, M. Rettig, C. K. Wong, L. True, A. Foye, D. Playdle, C. J. Ryan, P. Lara, K. N. Chi, V. Uzunangelov, A. Sokolov, Y. Newton, H. Beltran, F. Demichelis, M. A. Rubin, J. M. Stuart and E. J. Small		
E. Dardenne, H. Beltran, M. Benelli, K. Gayvert, A. Berger, L. Puca, J. Cyrta, A. Sboner, Z. Noorzad, T. MacDonald, C. Cheung, K. S. Yuen, D. Gao, Y. Chen, M. Eilers, J.-M. Mosquera, B. D. Robinson, O. Elemento, M. A. Rubin, F. Demichelis and D. S. Rickman	10.1016/j.ccell.2016.09.005	N-Myc Induces an EZH2-Mediated Transcriptional Program Driving Neuroendocrine Prostate Cancer
W. Wang and J. I. Epstein	10.1097/PAS.0b013e318058a96b	Small cell carcinoma of the prostate - A morphologic and immunohistochemical study of 95 cases
Y. J. Bang, F. Pirnia, W. G. Fang, W. K. Kang, O. Sartor, L. Whitesell, M. J. Ha, M. Tsokos, M. D. Sheahan, P. Nguyen, W. T. Niklinski, C. E. Myers and J. B. Trepel	10.1073/pnas.91.12.5330	Terminal neuroendocrine differentiation of human prostate carcinoma cells in response to increased intracellular cyclic AMP
B. Tetu, J. Y. Ro, A. G. Ayala, D. E. Johnson, C. J. Logothetis and N. G. Ordonez	10.1002/1097-0142(19870515)59:10<1803::Aid-cncr2820591019>3.0.Co;2-x	Small cell carcinoma of the prostate. Part I. A clinicopathologic study of 20 cases
J. K. Lee, J. W. Phillips, B. A. Smith, J. W. Park, T. Stoyanova, E. F. McCaffrey, R. Baertsch, A. Sokolov, J. G. Meyerowitz, C.	10.1016/j.ccell.2016.03.001	N-Myc Drives Neuroendocrine Prostate Cancer Initiated from Human Prostate Epithelial Cells

Mathis, D. Cheng, J. M. Stuart, K. M. Shokat, W. C. Gustafson, J. Huang and O. N. Witte		
D. Hirano, Y. Okada, S. Minei, Y. Takimoto and N. Nemoto	10.1016/j.eururo.2003.11.032	Neuroendocrine differentiation in hormone refractory prostate cancer following androgen deprivation therapy
P. A. Abrahamsson	10.1677/erc.0.0060503	Neuroendocrine cells in tumour growth of the prostate
Y. Qiu, D. Robinson, T. G. Pretlow and H. J. Kung	10.1073/pnas.95.7.3644	Etk/Bmx, a tyrosine kinase with a pleckstrin-homology domain, is an effector of phosphatidylinositol 3'-kinase and is involved in interleukin 6-induced neuroendocrine differentiation of prostate cancer cells

M. E. Cox, P. D. Deeble, S. Lakhani and S. J. Parsons		Acquisition of neuroendocrine characteristics by prostate tumor cells is reversible: Implications for prostate cancer progression
N. Vashchenko and P. A. Abrahamsson	10.1016/j.eururo.2004.09.007	Neuroendocrine differentiation in prostate cancer: Implications for new treatment modalities
N. Masumori, T. Z. Thomas, P. Chaurand, T. Case, M. Paul, S. Kasper, R. M. Caprioli, T. Tsukamoto, S. B. Shappell and R. J. Matusik		A probasin-large T antigen transgenic mouse line develops prostate adenocarcinoma and neuroendocrine carcinoma with metastatic potential
C. N. Papandreou, D. D. Daliani, P. F. Thall, S. M. Tu, X. M. Wang, A. Reyes, P. Troncoso and C. J. Logothetis	10.1200/jco.2002.12.065	Results of a phase II study with doxorubicin, etoposide, and cisplatin in patients with fully characterized small-cell carcinoma of the prostate

J. L. Bishop, D. Thaper, S. Vahid, A. Davies, K. Ketola, H. Kuruma, R. Jama, K. M. Nip, A. Angeles, F. Johnson, A. W. Wyatt, L. Fazli, M. E. Gleave, D. Lin, M. A. Rubin, C. C. Collins, Y. Wang, H. Beltran and A. Zoubeidi	10.1158/2159-8290.Cd-15-1263	The Master Neural Transcription Factor BRN2 Is an Androgen Receptor-Suppressed Driver of Neuroendocrine Differentiation in Prostate Cancer
A. G. Aprikian, C. Cordon-Cardo, W. R. Fair and V. E. Reuter	10.1002/1097-0142(19930615)71:12<3952::Aid-cncr2820711226>3.0.Co;2-x	Characterization of neuroendocrine differentiation in human benign prostate and prostatic adenocarcinoma
J. Qi, K. Nakayama, R. D. Cardiff, A. D. Borowsky, K. Kaul, R. Williams, S. Krajewski, D. Mercola, P. M. Carpenter, D. Bowtell and Z. e. A. Ronai	10.1016/j.ccr.2010.05.024	Siah2-Dependent Concerted Activity of HIF and FoxA2 Regulates Formation of Neuroendocrine Phenotype and Neuroendocrine Prostate Tumors
T.-C. Yuan, S. Veeramani and M.-F. Lin	10.1677/erc-07-0061	Neuroendocrine-like prostate cancer cells: Neuroendocrine transdifferentiation of prostate adenocarcinoma cells

A. H. Davies, H. Beltran and A. Zoubeidi	10.1038/nrurol.2018.22	Cellular plasticity and the neuroendocrine phenotype in prostate cancer
J. M. Mosquera, H. Beltran, K. Park, T. Y. MacDonald, B. D. Robinson, S. T. Tagawa, S. Perner, T. A. Bismar, A. Erbersdobler, R. Dhir, J. B. Nelson, D. M. Nanus and M. A. Rubin	10.1593/neo.121550	Concurrent AURKA and MYCN Gene Amplifications Are Harbingers of Lethal TreatmentRelated Neuroendocrine Prostate Cancer
T. Chiaverotti, S. S. Couto, A. Donjacour, J.-H. Mao, H. Nagase, R. D. Cardiff, G. R. Cunha and A. Balmain	10.2353/ajpath.2008.070602	Dissociation of epithelial and neuroendocrine carcinoma lineages in the transgenic adenocarcinoma of mouse prostate model of prostate cancer
S. Akamatsu, A. W. Wyatt, D. Lin, S. Lysakowski, F. Zhang, S. Kim, C. Tse, K. Wang, F. Mo, A. Haegert, S. Brahmbhatt, R. Bell, H. Adomat, Y. Kawai, H. Xue, X. Dong, L. Fazli, H. Tsai, T. L. Lotan, M. Kossai, J. M. Mosquera, M. A. Rubin, H. Beltran, A. Zoubeidi, Y. Wang, M. E. Gleave and C. C. Collins	10.1016/j.celrep.2015.07.012	The Placental Gene PEG10 Promotes Progression of Neuroendocrine Prostate Cancer

N. R. Mucci, G. Akdas, S. Manely and M. A. Rubin	10.1053/hp.2000.7295	Neuroendocrine expression in metastatic prostate cancer: Evaluation of high throughput tissue microarrays to detect heterogeneous protein expression
L. E. Littlepage, M. D. Sternlicht, N. Rougier, J. Phillips, E. Gallo, Y. Yu, K. Williams, A. Brenot, J. I. Gordon and Z. Werb	10.1158/0008-5472.Can-09-3515	Matrix Metalloproteinases Contribute Distinct Roles in Neuroendocrine Prostate Carcinogenesis, Metastasis, and Angiogenesis Progression
M. T. Spiotto and T. D. K. Chung	10.1002/(sici)1097-0045(20000215)42:3<186::Aid-pros4>3.0.Co;2-e	STAT3 mediates IL-6-induced neuroendocrine differentiation in prostate cancer cells
S. Terry and H. Beltran	10.3389/fonc.2014.00060	The many faces of neuroendocrine differentiation in prostate cancer progression
P. D. Deeble, D. J. Murphy, S. J. Parsons and M. E. Cox	10.1128/mcb.21.24.8471-8482.2001	Interleukin-6-and cyclic AMP-mediated signaling potentiates neuroendocrine differentiation of LNCaP prostate tumor cells
J. L. Yao, R. Madeb, P. Bourne, J. Lei, X. Yang, S. Tickoo, Z. Liu, D. Tan, L. Cheng, F. Hatem, J. Huang and P. A. di Sant' Agnese	10.1097/00000478-200606000-00005	Small cell carcinoma of the prostate: An immunohistochemical study

M. H. Weinstein, A. W. Partin, R. W. Veltri and J. I. Epstein	10.1016/s0046-8177(96)90398-6	Neuroendocrine differentiation in prostate cancer: enhanced prediction of progression after radical prostatectomy
F. Abbas, F. Civantos, P. Benedetto and M. S. Soloway	10.1016/s0090-4295(99)80290-8	Small cell carcinoma of the bladder and prostate
V. Parimi, R. Goyal, K. Poropatich and X. J. Yang		Neuroendocrine differentiation of prostate cancer: a review
T. Burchardt, M. Burchardt, M. W. Chen, Y. C. Cao, A. De la Taille, A. Shabsigh, O. Hayek, T. Dorai and R. Buttyan	10.1016/s0022-5347(05)68241-9	Transdifferentiation of prostate cancer cells to a neuroendocrine cell phenotype in vitro and in vivo
H. Bonkhoff		Neuroendocrine cells in benign and malignant prostate tissue: morphogenesis, proliferation, and androgen receptor status
P. Mariot, K. Vanoverberghe, N. Lalevee, M. F. Rossier and N. Prevarskaya	10.1074/jbc.M108754200	Overexpression of an alpha(1H) (Ca(v)3.2) T-type calcium channel during neuroendocrine differentiation of human prostate cancer cells
J. E. Oesterling, C. G. Hauzeur and G. M. Farrow	10.1016/s0022-5347(17)37390-1	Small cell anaplastic carcinoma of the prostate: a clinical, pathological and immunohistological study of 27 patients
A. V. Lapuk, C. Wu, A. W. Wyatt, A. McPherson, B. J. McConeghy, S. Brahmbhatt, F. Mo, A. Zoubeidi, S. Anderson, R. H. Bell, A. Haegert, R. Shukin, Y. Wang, L. Fazli, A. Hurtado-Coll, E. C. Jones, F. Hach, F. Hormozdiari, I.	10.1002/path.4047	From sequence to molecular pathology, and a mechanism driving the neuroendocrine phenotype in prostate cancer

Hajirasouliha, P. C. Boutros, R. G. Bristow, Y. Zhao, M. A. Marra, A. Fanjul, C. A. Maher, A. M. Chinnaiyan, M. A. Rubin, H. Beltran, S. C. Sahinalp, M. E. Gleave, S. V. Volik and C. C. Collins		
R. Nadal, M. Schweizer, O. N. Kryvenko, J. I. Epstein and M. A. Eisenberger	10.1038/nrurol.2014.21	Small cell carcinoma of the prostate
M. E. Wright, M. J. Tsai and R. Aebersold	10.1210/me.2003-0031	Androgen receptor represses the neuroendocrine transdifferentiation process in prostate cancer cells
R. M. Sainz, J. C. Mayo, D. X. Tan, J. Leon, L. Manchester and R. J. Reiter	10.1002/pros.20155	Melatonin reduces prostate cancer cell growth leading to neuroendocrine differentiation via a receptor and PKA independent mechanism
E. M. Garabedian, P. A. Humphrey and J. I. Gordon	10.1073/pnas.95.26.15382	A transgenic mouse model of metastatic prostate cancer originating from neuroendocrine cells

J. S. Palmgren, S. S. Karavadia and M. R. Wakefield	10.1053/j.seminoncol.2006.10.026	Unusual and underappreciated: Small cell carcinoma of the prostate
Y. Sun, J. Niu and J. Huang		Neuroendocrine differentiation in prostate cancer
J. Y. Ro, B. Tetu, A. G. Ayala and N. G. Ordonez	10.1002/1097-0142(19870301)59:5<977::Aid-cncr2820590521>3.0.Co;2-g	Small cell carcinoma of the prostate. II. Immunohistochemical and electron microscopic studies of 18 cases
J. L. Krijnen, P. J. Janssen, J. A. Ruizeveld de Winter, H. van Krimpen, F. H. Schroder and T. H. van der Kwast	10.1007/bf00268938	Do neuroendocrine cells in human prostate cancer express androgen receptor?
K. Vanoverberghe, F. Vanden Abeele, P. Mariot, G. Lepage, M. Roudbaraki, J. L. Bonnal, B. Mauroy, Y. Shuba, R. Skryma and N. Prevarskaya	10.1038/sj.cdd.4401375	Ca2+ homeostasis and apoptotic resistance of neuroendocrine-differentiated prostate cancer cells
T. C. Yuan, S. Veeramani, F. F. Lin, D. Kondrikou, S. Zelivianski, T. Igawa, D. Karan, S. K. Batra and M. F. Lin	10.1677/erc.1.01043	Androgen deprivation induces human prostate epithelial neuroendocrine differentiation of androgen-sensitive LNCaP cells

M. Borre, B. Nerstrom and J. Overgaard		Association between immunohistochemical expression of vascular endothelial growth factor (VEGF), VEGF-expressing neuroendocrine-differentiated tumor cells, and outcome in prostate cancer patients subjected to watchful waiting
A. Berruti, L. Dogliotti, A. Mosca, M. Bellina, M. Mari, M. Torta, R. Tarabuzzi, E. Bollito, D. Fontana and A. Angeli	10.1002/1097-0142(20000601)88:11<2590::Aid-cncr23>3.0.Co;2-d	Circulating neuroendocrine markers in patients with prostate carcinoma
P. Haag, J. Bektic, G. Bartsch, H. Mocker and I. E. Eder	10.1016/j.jsbmb.2005.04.029	Androgen receptor down regulation by small interference RNA induces cell growth inhibition in androgen sensitive as well as in androgen independent prostate cancer cells
N. Z. Xing, J. Q. Qian, D. Bostwick, E. Bergstralh and C. Y. F. Young	10.1002/pros.1076	Neuroendocrine cells in human prostate over-express the anti-apoptosis protein survivin
P. Uysal-Onganer, Y. Kawano, M. Caro, M. M. Walker, S. Diez, R. S. Darrington, J. Waxman and R. M. Kypta	10.1186/1476-4598-9-55	Wnt-11 promotes neuroendocrine-like differentiation, survival and migration of prostate cancer cells
A. J. Evans, P. A. Humphrey, J. Belani, T. H. van der Kwast and J. R. Srigley	10.1097/00000478-200606000-00003	Large cell neuroendocrine carcinoma of prostate - A clinicopathologic summary of 7 cases of a rare manifestation of advanced prostate cancer
H. Beltran, C. Oromendia, D. C. Danila, B. Montgomery, C. Hoimes, R. Z. Szmulewitz, U. Vaishampayan, A. J. Armstrong, M. Stein, J. Pinski, J. M. Mosquera, V. Sailer, R.	10.1158/1078-0432.Ccr-18-1912	A Phase II Trial of the Aurora Kinase A Inhibitor Alisertib for Patients with Castration-resistant and Neuroendocrine Prostate Cancer: Efficacy and Biomarkers

Bareja, A. Romanel, N. Gumpeni, A. Sboner, E. Dardenne, L. Puca, D. Prandi, M. A. Rubin, H. I. Scher, D. S. Rickman, F. Demichelis, D. M. Nanus, K. V. Ballman and S. T. Tagawa		
X. Zhang, I. M. Coleman, L. G. Brown, L. D. True, L. Kollath, J. M. Lucas, H.-M. Lam, R. Dumpit, E. Corey, L. Chery, B. Lakely, C. S. Higano, B. Montgomery, M. Roudier, P. H. Lange, P. S. Nelson, R. L. Vessella and C. Morrissey	10.1158/1078-0432.Ccr-15-0157	SRRM4 Expression and the Loss of REST Activity May Promote the Emergence of the Neuroendocrine Phenotype in Castration-Resistant Prostate Cancer
Y. Li, N. Donmez, C. Sahinalp, N. Xie, Y. Wang, H. Xue, F. Mo, H. Beltran, M. Gleave, Y. Wang, C. Collins and X. Dong	10.1016/j.eururo.2016.04.028	SRRM4 Drives Neuroendocrine Transdifferentiation of Prostate Adenocarcinoma Under Androgen Receptor Pathway Inhibition
R. Aggarwal, T. Zhang, E. J. Small and A. J. Armstrong	10.6004/jnccn.2014.0073	Neuroendocrine Prostate Cancer: Subtypes, Biology, and Clinical Outcomes
H. Bonkhoff	10.1093/annonc/12.suppl_2.S141	Neuroendocrine differentiation in human prostate cancer. Morphogenesis, proliferation and androgen receptor status

A. Komiya, H. Suzuki, T. Imamoto, N. Kamiya, N. Nihei, Y. Naya, T. Ichikawa and H. Fuse	10.1111/j.1442-2042.2008.02175.x	Neuroendocrine differentiation in the progression of prostate cancer
A. Angelsen, U. Syversen, O. A. Haugen, M. Stridsberg, O. K. Mjolnerod and H. L. Waldum		Neuroendocrine differentiation in carcinomas of the prostate: do neuroendocrine serum markers reflect immunohistochemical findings
M. E. Cox, P. D. Deeble, E. A. Bissonette and S. J. Parsons	10.1074/jbc.275.18.13812	Activated 3 ',5 '-cyclic AMP-dependent protein kinase is sufficient to induce neuroendocrine-like differentiation of the LNCaP prostate tumor cell line
V. Tzelepi, J. Zhang, J.-F. Lu, B. Kleb, G. Wu, X. Wan, A. Hoang, E. Efstathiou, K. Sircar, N. M. Navone, P. Troncoso, S. Liang, C. J. Logothetis, S. N. Maity and A. M. Aparicio	10.1158/1078-0432.Ccr-11-1867	Modeling a Lethal Prostate Cancer Variant with Small-Cell Carcinoma Features
D. S. Schron, T. Gipson and G. Mendelsohn	10.1002/1097-0142(19840601)53:11<2478::Aid-cncr2820531119>3.0.Co;2-q	The histogenesis of small cell carcinoma of the prostate. An immunohistochemical study
H. Beltran, A. Jendrisak, M. Landers, J. M. Mosquera, M. Kossai, J. Louw, R. Krupa, R. P. Graf, N. A. Schreiber, D. M. Nanus, S. T. Tagawa, D. Marrinucci, R. Dittamore and H. I. Scher	10.1158/1078-0432.Ccr-15-0137	The Initial Detection and Partial Characterization of Circulating Tumor Cells in Neuroendocrine Prostate Cancer

M. A. Noordzij, T. H. van der Kwast, G. J. van Steenbrugge, W. J. Hop and F. H. Schroder	10.1002/ijc.2910620304	The prognostic influence of neuroendocrine cells in prostate cancer: results of a long-term follow-up study with patients treated by radical prostatectomy
V. Conteduca, C. Oromendia, K. W. Eng, R. Bareja, M. Sigouros, A. Molina, B. M. Faltas, A. Sboner, J. M. Mosquera, O. Elemento, D. M. Nanus, S. T. Tagawa, K. V. Ballman and H. Beltran	10.1016/j.ejca.2019.08.011	Clinical features of neuroendocrine prostate cancer
J. Luo, K. Wang, S. Yeh, Y. Sun, L. Liang, Y. Xiao, W. Xu, Y. Niu, L. Cheng, S. N. Maity, R. Jiang and C. Chang	10.1038/s41467-019-09784-9	LncRNA-p21 alters the antiandrogen enzalutamide-induced prostate cancer neuroendocrine differentiation via modulating the EZH2/STAT3 signaling
X. Z. Yang, M. W. Chen, S. Terry, F. Vacherot, D. K. Chopin, D. L. Bemis, J. Kitajewski, N. C. Benson, Y. L. Guo and R. Buttyan	10.1158/0008-5472.Can-05-0162	A human- and male-specific protocadherin that acts through the Wnt signaling pathway to induce neuroendocrine transdifferentiation of prostate cancer cells
G. S. Palapattu, C. Wu, C. R. Silvers, H. B. Martin, K. Williams, L. Salamone, T. Bushnell, L.-S. Huang, Q. Yang and J. Huang	10.1002/pros.20928	Selective Expression of CD44, a Putative Prostate Cancer Stem Cell Marker, in Neuroendocrine Tumor Cells of Human Prostate Cancer
J. L. Krijnen, J. F. Bogdanowicz, C. A. Seldenrijk, P. G. Mulder and T. H. van der Kwast	10.1097/00005392-199707000-00054	The prognostic value of neuroendocrine differentiation in adenocarcinoma of the prostate in relation to progression of disease after endocrine therapy

H. Guo, X. Ci, M. Ahmed, J. T. Hua, F. Soares, D. Lin, L. Puca, A. Vosoughi, H. Xue, E. Li, P. Su, S. Chen, N. Tran, Y. Liang, Y. Zhang, X. Xu, J. Xu, A. V. Sheahan, W. Ba-Alawi, S. Zhang, O. Mahamud, R. N. Vellanki, M. Gleave, R. G. Bristow, B. Haibe-Kains, J. T. Poirier, C. M. Rudin, M.-S. Tsao, B. G. Wouters, L. Fazli, F. Y. Feng, L. Ellis, T. van der Kwast, A. Berlin, M. Koritzinsky, P. C. Boutros, A. Zoubeidi, H. Beltran, Y. Wang and H. H. He	10.1038/s41467-018-08133-6	ONECUT2 is a driver of neuroendocrine prostate cancer
R. Shen, T. Dorai, M. Szaboles, A. E. Katz, C. A. Olsson and R. Buttyan	10.1016/s1078-1439(97)00039-2	Transdifferentiation of cultured human prostate cancer cells to a neuroendocrine cell phenotype in a hormone-depleted medium
P. Pittoni, C. Tripodo, S. Piconese, G. Mauri, M. Parenza, A. Rigoni, S. Sangaletti and M. P. Colombo	10.1158/0008-5472.Can-11-1637	Mast Cell Targeting Hampers Prostate Adenocarcinoma Development but Promotes the Occurrence of Highly Malignant Neuroendocrine Cancers
C.-D. Hu, R. Choo and J. Huang	10.3389/fonc.2015.00090	Neuroendocrine differentiation in prostate cancer: a mechanism of radioresistance and treatment failure
J. Huang, J. L. Yao, A. di Sant'Agnese, Q. Yang, P. A. Bourne and Y. Na	10.1002/pros.20434	Immunohistochemical characterization of neuroendocrine cells in prostate cancer

J. T. Huang, J. L. Yao, L. Zhang, P. A. Bourne, A. M. Quinn, P. A. di Sant'Agnese and J. E. Reeder	10.1016/s0002-9440(10)62490-x	Differential expression of interleukin-8 and its receptors in the neuroendocrine and non-neuroendocrine compartments of prostate cancer
X. Deng, H. Liu, J. Huang, L. Cheng, E. T. Keller, S. J. Parsons and C.-D. Hu	10.1158/0008-5472.Can-08-2229	Ionizing Radiation Induces Prostate Cancer Neuroendocrine Differentiation through Interplay of CREB and ATF2: Implications for Disease Progression
P. A. Abrahamsson		Neuroendocrine differentiation and hormone-refractory prostate cancer
A. Flechon, D. Pouessel, C. Ferlay, D. Perol, P. Beuzeboc, G. Gravis, F. Joly, S. Oudard, G. Deplanque, S. Zanetta, P. Fargeot, F. Priou, J. P. Droz and S. Culine	10.1093/annonc/mdr004	Phase II study of carboplatin and etoposide in patients with anaplastic progressive metastatic castration-resistant prostate cancer (mCRPC) with or without neuroendocrine differentiation: results of the French Genito-Urinary Tumor Group (GETUG) P01 trial
S. Zelivianski, M. Verni, C. Moore, D. Kondrikov, R. Taylor and M. F. Lin	10.1016/s0167-4889(01)00087-8	Multipathways for transdifferentiation of human prostate cancer cells into neuroendocrine-like phenotype

M. Reina-Campos, J. F. Linares, A. Duran, T. Cordes, A. L'Hermitte, M. G. Badur, M. S. Bhangoo, P. K. Thorson, A. Richards, T. Rooslid, D. C. Garcia-Olmo, S. Y. Nam-Cha, A. S. Salinas-Sanchez, K. Eng, H. Beltran, D. A. Scott, C. M. Metallo, J. Moscat and M. T. Diaz-Meco	10.1016/j.ccell.2019.01.018	Increased Serine and One-Carbon Pathway Metabolism by PKC lambda/l Deficiency Promotes Neuroendocrine Prostate Cancer
Y. Hu, J. E. Ippolito, E. M. Garabedian, P. A. Humphrey and J. I. Gordon	10.1074/jbc.M205784200	Molecular characterization of a metastatic neuroendocrine cell cancer arising in the prostates of transgenic mice
D. G. Bostwick, J. Q. Qian, A. Pacelli, H. Zincke, M. Blute, E. J. Bergstralh, J. M. Slezak and L. Cheng	10.1016/s0022-5347(05)64626-5	Neuroendocrine expression in node positive prostate cancer: Correlation with systemic progression and patient survival
P. Sotomayor, A. Godoy, G. J. Smith and W. J. Huss	10.1002/pros.20895	Oct4A is Expressed by a Subpopulation of Prostate Neuroendocrine Cells
F. Crea, E. Venalainen, X. Ci, H. Cheng, L. Pikor, A. Parolia, H. Xue, N. R. N. Saidy, D. Lin, W. Lam, C. Collins and Y. Wang	10.2217/epi.16.6	The role of epigenetics and long noncoding RNA MIAT in neuroendocrine prostate cancer
G. Ahlgren, K. Pedersen, S. Lundberg, G. Aus, J. Hugosson and P. A. Abrahamsson	10.1002/(sici)1097-0045(20000301)42:4<274::Aid-pros4>3.0.Co;2-r	Regressive changes and neuroendocrine differentiation in prostate cancer after neoadjuvant hormonal treatment

C. C. Guo, J. Y. Dancer, Y. Wang, A. Aparicio, N. M. Navone, P. Troncoso and B. A. Czerniak	10.1016/j.humpath.2010.05.026	TMPRSS2-ERG gene fusion in small cell carcinoma of the prostate
C. Wu and J. Huang	10.1074/jbc.M608487200	Phosphatidylinositol 3-kinase-AKT-mammalian target of rapamycin pathway is essential for neuroendocrine differentiation of prostate cancer
S. O. Lee, J. Y. Chun, N. Nadiminty, W. Lou and A. C. Gao	10.1002/pros.20553	Interleukin-6 undergoes transition from growth inhibitor associated with neuroendocrine differentiation to stimulator accompanied by androgen receptor activation during LNCaP prostate cancer cell progression
P. A. di Sant'Agnese	10.1016/s0090-4295(98)00064-8	Neuroendocrine cells of the prostate and neuroendocrine differentiation in prostatic carcinoma: a review of morphologic aspects
S. Akamatsu, T. Inoue, O. Ogawa and M. E. Gleave	10.1111/iju.13526	Clinical and molecular features of treatment-related neuroendocrine prostate cancer
N. H. Segal, R. J. Cohen, Z. Haffejee and N. Savage		BCL-2 proto-oncogene expression in prostate cancer and its relationship to the prostatic neuroendocrine cell
P. E. Spiess, C. A. Pettaway, F. Vakar-Lopez, W. Kassouf, X. Wang, J. E. Busby, K.-A. Do, R. Davuluri and N. M. Tannir	10.1002/cncr.22971	Treatment outcomes of small cell carcinoma of the prostate - A single-center study
G. P. Amorino and S. J. Parsons	10.1615/CritRevEukaryotGeneExpr.v14.i4.40	Neuroendocrine cells in prostate cancer

F. Gackiere, G. Bidaux, P. Delcourt, F. Van Coppenolle, M. Katsogiannou, E. Dewailly, A. Bavencoffe, M. T. Van Chuoi-Mariot, B. Mauroy, N. Prevarskaya and P. Mariot	10.1074/jbc.M707159200	CaV3.2 T-type calcium channels are involved in calcium-dependent secretion of neuroendocrine prostate cancer cells
J. Kim, H. Jin, J. C. Zhao, Y. A. Yang, Y. Li, X. Yang, X. Dong and J. Yu	10.1038/onc.2017.50	FOXA1 inhibits prostate cancer neuroendocrine differentiation
E. C. Nelson, A. J. Cambio, J. C. Yang, J. H. Ok, P. N. Lara, Jr. and C. P. Evans	10.1038/sj.pcan.4500922	Clinical implications of neuroendocrine differentiation in prostate cancer
P.-L. Clermont, D. Lin, F. Crea, R. Wu, H. Xue, Y. Wang, K. L. Thu, W. L. Lam, C. C. Collins, Y. Wang and C. D. Helgason	10.1186/s13148-015-0074-4	Polycomb-mediated silencing in neuroendocrine prostate cancer
